# Investigation of the Ligand Exchange Process on Gold Nanorods by Using Laser Desorption/Ionization Time-of-Flight Mass Spectrometry

**DOI:** 10.3390/ma15134406

**Published:** 2022-06-22

**Authors:** Seung-Woo Kim, Young Won Kim, Tae Hoon Seo, Young-Kwan Kim

**Affiliations:** 1 Department of Chemistry, Dongguk University, 30 Pildong-ro, Jung-gu, Seoul 04620, Korea; swkim94@dgu.ac.kr; 2 Green Energy & Nano Technology R&D Group, Korea Institute of Industrial Technology, 6 Cheomdan-gwagiro 208-gil, Buk-gu, Gwangju 61012, Korea; ywkim@kitech.re.kr

**Keywords:** gold nanorods, laser desorption/ionization, ligand exchange, surface plasmon resonance, surface modification

## Abstract

The ligand exchange process on gold nanorods (Au NRs) was explored by using laser desorption/ionization time-of-flight mass spectrometry (LDI-TOF-MS). Cetyltrimethylammonium bromide (CTAB) adsorbed on Au NRs was replaced with alkanethiol derivatives presenting different functional groups. The ligand exchange process was investigated under various conditions, such as in the presence of different functional groups in the ligands and with different concentrations of CTAB. The ligand-exchanged Au NRs were characterized by using a combination of UV–Vis spectroscopy and LDI-TOF-MS. Based on the results, it was revealed that LDI-TOF-MS analysis can provide crucial and distinct information about the degree of ligand exchange on Au NRs.

## 1. Introduction

Gold nanorods (Au NRs) have attracted much attention from various research fields owing to their unique optical properties, as they exhibit two localized surface plasmon resonance (LSPR) modes in their transverse and longitudinal directions, resulting in strong absorption and scattering in the visible and near-infrared (Vis–NIR) region [1]. Those optical properties make Au NRs promising agents for biosensing and bioimaging [2,3]. The longitudinal LSPR mode of Au NRs is particularly important because it can fit the biological window for bioimaging and phototherapy [4]. In spite of their strong potential, biological applications of Au NRs have been restricted by their high cytotoxicity which originates from the residual surface capping agent, cetyltrimethylammonium bromide (CTAB) [5]. The typical synthetic method of Au NRs is seed-mediated growth utilizing small Au seeds for anisotropic growth in the presence of CTAB, which preferentially binds to the {110} crystalline facet of Au seeds and thus leads to specific growth on this {100} facet [6].

Therefore, surface modification of Au NRs is a prerequisite for biological applications, and various molecules such as polyelectrolytes [7], inorganic ligands [8], lipids [9], and thiol derivatives [10,11,12,13] have been harnessed for the promotion of ligand exchange as replacements for CTAB. Among those molecules, thiol derivatives are particularly important due to their strong affinity leading to a stable Au-S interaction [10,11,12,13]. Although the ligand exchange of Au NRs with thiol derivatives has been widely investigated, it is still an urgent issue to optimize the ligand exchange process of Au NRs for their successful biological application.

There is a demand for simple and precise analytical tools for the systematic examination of the ligand exchange process on Au NRs, and thus various spectroscopic techniques have been applied to analyze the ligand exchange process [14,15]. However, UV–Vis spectroscopy can provide indirect information based on the shift of LSPR bands, and FT-IR spectroscopy is not sensitive and requires a large amount of sample [15]. In addition, Raman spectroscopy has shown limited applications for several ligands that have certain structural features [9]. X-ray photoelectron spectroscopy is also a powerful tool but cannot provide information on the chemical structure of thiol ligands at the molecular-level [9,15].

Matrix-assisted laser desorption/ionization time-of-flight mass spectrometry (MALDI-TOF-MS) is a soft ionization technique that has been extensively harnessed for mass spectrometric analysis of high-molecular-weight compounds such as synthetic polymers and proteins without fragmentation [16]. Despite its many advantages, MALDI-TOF-MS cannot be directly applied to mass spectrometric analysis of low-molecular-weight compounds owing to matrix interference in the low mass region (<500 Da) [17]. Many efforts have been devoted to overcoming this crucial problem by using metallic, semiconductor, and carbon nanomaterials [17]. These nanomaterials have a large surface area, high UV absorption capacity, and photo-thermal conversion properties which are requirements for efficient matrix-free laser desorption/ionization time-of-flight mass spectrometry (LDI-TOF-MS). Especially, gold nanoparticles (Au NPs) have been widely investigated for LDI-TOF-MS analysis of small molecules due to their high efficiency derived from high photothermal conversion through electronic interband and/or intraband transitions, which could promote the laser-induced heating and phase transition process of NPs [18]. Owing to its high resolution, sensitivity, and small consumption of sample, it has been also reported that the surface-adsorbed molecules on Au NPs can be detected with LDI-TOF-MS, and thus this technique has drawn much attention as a promising analytical strategy for the surface characterization of Au NPs [19,20,21]. Dopamine and thiol ligands have been successfully detected on magnetic [22], semiconductor [23], and metal nanoparticles (NPs) by using LDI-TOF-MS [21]. Thus, the ligand exchange process of these NPs has been extensively investigated with LDI-TOF-MS [21,22,23]. However, no reports have been published on the investigation of ligand exchange, and only a few reports are present in the literature on the characterization of elemental composition and CTAB on the surface of Au NRs with LDI-TOF-MS [24,25].

**Figure 1:**
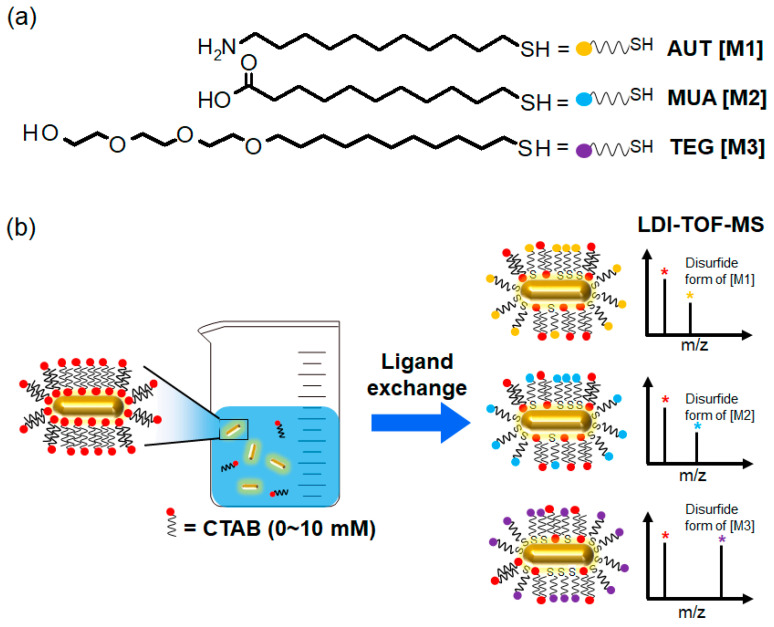
(**a**) Structures of the thiol derivatives AUT, MUA, and TEG. (**b**) Ligand exchange of Au NRs with the thiol derivatives AUT, MUA, and TEG in the presence of different concentrations of CTAB and subsequent surface characterization of the Au NRs by using LDI-TOF-MS. The red, yellow, blue, and purple asterisks in LDI-TOF-MS spectra indicate CTAB and disulfide form adducts of M1, M2, and M3, respectively.

Herein, we investigated the concentration effect of free CTAB on the ligand exchange process of Au NRs by using a combination of UV–Vis spectroscopy and LDI-TOF-MS (Figure 1). Au NRs were synthesized by the seed-mediated growth method [6] and characterized with conventional tools such as UV–Vis–NIR spectroscopy and scanning electron microscopy (SEM). Then, CTAB on Au NRs was replaced with thiol derivatives such as 11-amino-1-undecanethiol (AUT), 11-mercaptoundecanoic acid (MUA), and (11-mercaptoundecyl)tri(ethylene glycol) (TEG), which contain primary amine, carboxylic acid, and hydroxyl functional groups as their end groups, respectively. The Au NRs surface-modified under various conditions were characterized with UV–Vis spectroscopy and LDI-TOF-MS to clearly estimate the degree of their ligand exchange.

## 2. Materials and Methods

### 2.1. Materials

Hydrogen tetrachloroaurate(III) hydrate was purchased from Kojima chemicals (Japan). Cetyl trimethyl ammonium bromide (CTAB) was purchased from Across (New Jersey, USA). Ethanol was purchased from Merck (Darmstadt, Germany). AUT, MUA, TEG, silver nitrate, ascorbic acid and other reagents were purchased from Sigma-Aldrich (St. Louis, MO, USA).

### 2.2. Synthesis of Au NRs

Au NRs were synthesized by the seed-mediated growth process with minor modification. Au seeds were prepared by adding 250 μL of a 10 μM solution of HAuCl_4_ ∙ 3H_2_O to 7.5 mL of a 100 mM CTAB solution in a glass vial. The mixture was gently shaken, and its color became brown-yellow. Then, 600 μL of a 10 mM ice-cooled NaBH_4_ solution was added to the mixture, and the mixture was gently shaken for 2 min. After reaction, the color of the mixture changed to pale brown. The concentration of the prepared Au seeds was 21.7 nM, and the prepared Au seeds were kept at 25 °C at room temperature for 6 h before use. The growth solution was prepared by adding 2 mL of a 10 μM solution of HAuCl_4_∙3H_2_O and 300 μL of 10 mM AgNO_3_ to 47.5 mL of the 100 mM CTAB solution. The mixture was gently shaken, and its color was bright brown-yellow. Then, 320 μL of a 100 mM ascorbic acid solution was added to the mixture. After the addition, the color of the mixture gradually disappeared. Finally, 100 μL of the Au seed solution was added to the growth solution, and the mixture was gently shaken for 10 s and kept overnight at 25 °C without agitation. Au NRs were centrifuged at 12,857 rcf and re-suspended into water, and this process was repeated 2 times for the removal of excess CTAB. This centrifugation and re-suspension steps were carried out carefully to minimize the loss of Au NRs which can change the concentration of Au NRs. Based on the hypothesis that all the added Au seeds were converted into Au NRs, the final concentration of Au NRs was 43.2 pM. The diameter, length, and aspect ratio of the Au NRs were 25 ± 2 nm, 50 ± 2 nm and 2.1 ± 0.2, respectively.

### 2.3. Ligand Exchange of Au NRs

For the preparation of Au NRs with different concentrations of CTAB, the Au NRs were centrifuged at 12,857 rcf and re-dispersed in pure water and in 1 mM and 10 mM aqueous solutions of CTAB. Then, 1 mL of the Au NR suspensions with varying concentrations of CTAB was mixed with 100 μL of 1 mM ethanolic solutions of AUT, MUA, and TEG. The mixtures were incubated with gentle shaking for 12 h. After incubation, the mixtures were centrifuged at 12,857 rcf and re-suspended in water 3 times for the removal of excess thiol derivatives. This process was also carefully performed to minimize undesired concentration changes of Au NRs. We assumed that the concentration of Au NRs was not significantly changed unless gold chunks were precipitated or the suspension of the Au NRs became transparent.

### 2.4. Characterization

UV–Vis spectra were recorded by using a Cary 50 UV–Vis spectrophotometer (Varian, Belrose, Australia). SEM images of the Au NRs were obtained using an S4800 (HITACHI, Tokyo, Japan). LDI-TOF-MS analysis was carried out by using IDSys (ASTA, Suwon, Korea) with a 343 nm Nb:YAG laser having a pulse rate of 1 kHz and a laser spot diameter of 50 μm. The accelerating voltage was 18 kV in positive ionization mode. For LDI-TOF-MS analysis, 1 μL of the aqueous suspensions of the surface-modified Au NRs with AUT, MUA, and TEG was spotted on a stainless-steel target plate, dried under ambient conditions and subjected to LDI-TOF-MS analysis in positive ionization mode.

## 3. Results and Discussion

**Figure 2:**
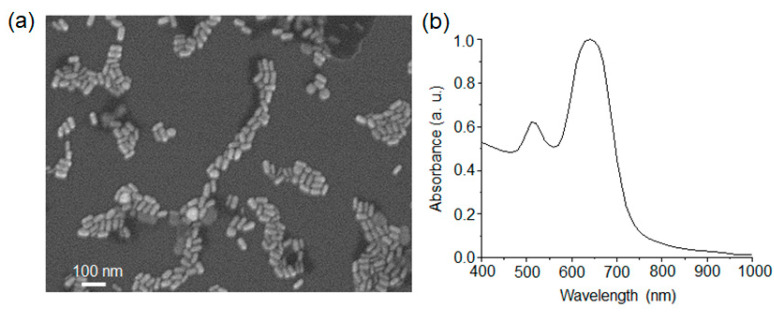
(**a**) SEM image and (**b**) UV–Vis spectrum of the Au NRs.

The synthesized Au NRs showed an average diameter, length, and aspect ratio of 25 ± 2 nm, 50 ± 2 nm and 2.1 ± 0.2, respectively (Figure 2a). Their UV–Vis spectrum exhibited two typical absorption peaks around 510 nm and 640 nm, which originated from their transverse and longitudinal plasmon resonance modes, respectively (Figure 2b). The absorption properties are typical plasmonic characteristics of Au NRs, which reflect their anisotropic shape. Then, the surface of the Au NRs was modified with the thiol derivatives AUT, MUA, and TEG which are representative positive and negatively charged thiols and neutral hydrophilic ligands. It is noteworthy that ligand exchange was carried out with a different amount of free CTAB to explore the effect of its concentration. The synthesized Au NRs were centrifuged and washed with water twice and were finally dispersed in distilled water and 1 mM and 10 mM aqueous solutions of CTAB. The ligand exchange process was carried out by the addition of the thiol ligands to the dispersed Au NRs having different amounts of free CTAB.

**Figure 3:**
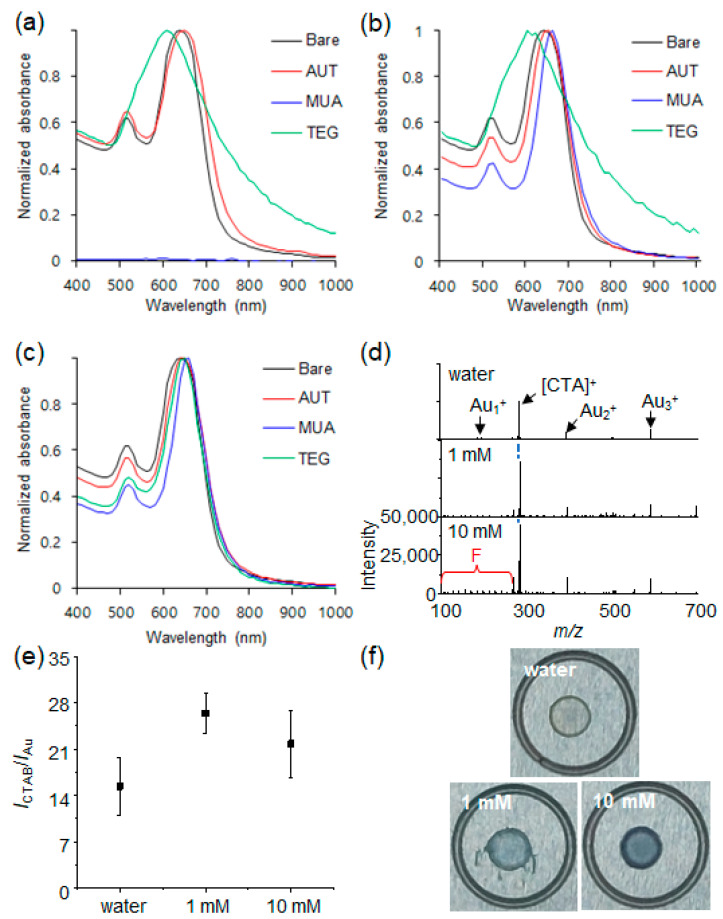
UV–Vis spectra of AUT-, MUA-, and TEG-modified Au NRs in (**a**) water, (**b**) 1 mM CTAB, and (**c**) 10 mM CTAB. (**d**) LDI-TOF-MS spectra of the modified Au NRs with different concentration of free CTAB. (**e**) The intensity ratio of the mass peaks from [CTA]^+^ and Au cluster ions obtained from Au NRs re-dispersed in water and 1 mM and 10 mM CTAB solutions. (**f**) Photographs of the spots formed on a target plate by evaporation of a single droplet of the re-dispersed Au NRs in water and 1 mM and 10 mM CTAB solutions.

**Figure 4:**
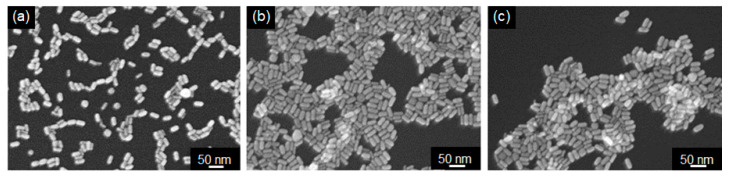
SEM images of Au NRs modified with (**a**) AUT, (**b**) MUA, (**c**) and TEG in 1 mM free CTAB.

After ligand exchange of Au NRs in water, AUT led to a slight red shift of the Au NRs longitudinal mode to 650 nm, without changes to their transverse mode (Figure 3a). The ligand exchange of Au NRs with TEG ligand caused a substantial change of the absorption spectrum. The absorption peaks derived from the transverse and longitudinal modes of Au NRs merged at 610 nm, which indicated the neutral ligand resulted in a partial aggregation of the Au NRs (Figure 3a). By stark contrast, the MUA ligand led to severe aggregation of Au NRs under equal ligand exchange conditions, and thus the absorption signal could not be obtained (Figure 3a). These results imply that the characteristics of the ligands should be considered for successful ligand exchange of Au NRs, and ligand exchange with neutral and negatively charged ligands requires an appropriate amount of free CTAB. The ligand exchange of Au NRs with thiol ligands was then conducted in a 1 mM aqueous solution of CTAB. The longitudinal mode of Au NRs was equally red-shifted to 650 nm with the AUT ligand, and this red shift matched with that obtained with no additional CTAB (Figure 3b). Likewise, the absorption features acquired with TEG were similar to those observed with AUT (Figure 3b). These results suggested that the red shift of the longitudinal mode of Au NRs was not affected by the concentration of free CTAB in the presence of AUT and TEG. Interestingly, aggregation of Au NRs did not occur by ligand exchange with MUA in these conditions, and thus the longitudinal mode of Au NRs was red-shifted to 660 nm, a larger shift than that caused by AUT (Figure 3b). This red shift indicated the ligand exchange of Au NRs was successfully carried out with MUA. It is obvious that the presence of free CTAB critically influenced the ligand exchange process of Au NRs with negatively charged thiol ligands. Then, ligand exchange was carried out in a 10 mM aqueous solution of CTAB. The red-shifted longitudinal mode of Au NRs was equally observed at 650 nm and 660 nm with AUT and MUA, respectively (Figure 3c). This similar red shift implied that the increase in the concentration of free CTAB from 1 mM to 10 mM did not affect the ligand exchange process of Au NRs with AUT and MUA. On the other hand, the ligand exchange of Au NRs with TEG led to drastic changes of Au NRs optical properties, which showed their clearly resolved transverse and longitudinal modes at 520 nm and 650 nm (Figure 3c). This result showed that the ligand exchange of Au NRs with TEG successfully occurred in this condition without NRs partial aggregation. These UV–Vis spectroscopic analyses indicated that the ligand exchange process of Au NRs was substantially affected by the concentration of free CTAB (Figure 3). The SEM images of Au NRs modified with AUT, MUA, and TEG in 1 mM free CTAB showed that the morphology of Au NRs was not changed during the ligand exchange process, implying the changes of their absorption spectrum were solely derived from surface modification (Figure 4). Based on the UV–Vis spectroscopic analysis, as the concentration of CTAB increased, the surface of Au NRs was efficiently modified with the thiol derivatives through the ligand exchange process. Considering that free CTAB can compete with thiol derivatives, the ligand exchange rate might diminish with the increase of CTAB concentration, but UV–Vis spectroscopy revealed opposite results.

**Figure 5:**
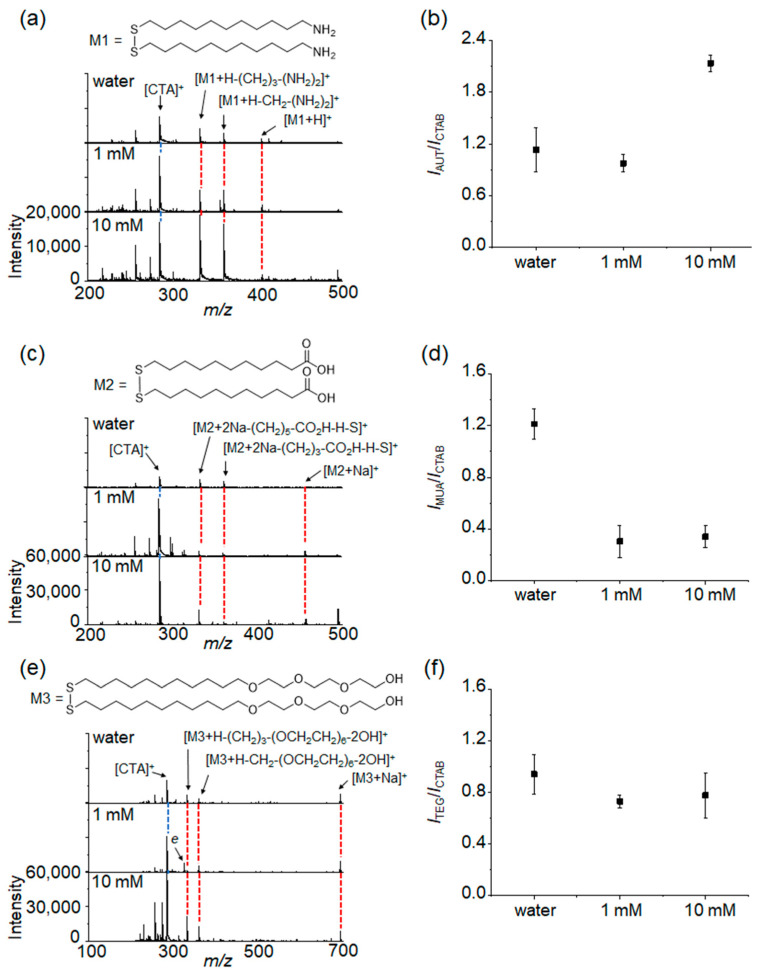
(**a**) LDI-TOF-MS spectra of ligand-exchanged Au NRs with (**a**) AUT, (**c**) MUA, and (**e**) TEG and the relative intensity ratios of the mass peaks derived from the residual CTAB and thiol ligands such as (**b**) AUT, (**d**) MUA, and (**f**) TEG on the modified Au NRs prepared in water and 1 mM and 10 mM CTAB. The average *I_ligand_*/*I_CTAB_* ratios were obtained from at least five different measurements.

To further examine the ligand exchange efficiency of Au NRs with thiol derivatives, the Au NRs were then characterized with LDI-TOF-MS. The synthesized Au NRs exhibited typical gold cluster ion peaks at *m*/*z* 196 [Au_1_]^+^, 393 [Au_2_]^+^, and 590 [Au_3_]^+^ and a peak at *m*/*z* 283 derived from cetyltrimethylammonium ion [CTA]^+^ [25]. As CTAB was strongly adsorbed on the surface of Au NRs, those mass peaks were still detected without additional CTAB after the washing step. The intensity ratio of the mass peaks (*I_CTAB_/I_Au cluster_*) from [CTA]^+^ and Au cluster ions such as Au^+^, Au_2_^+^, and Au_3_^+^ was calculated as 15.3 ± 4.3 for the Au NRs re-dispersed in water (Figure 3d,e). The *I_CTAB_/I_Au cluster_* of the Au NRs re-dispersed in 1 mM CTAB increased to 26.4 ± 3.0 and slightly decreased to 21.8 ± 5.0 after re-dispersion in 10 mM CTAB (Figure 3d,e). This result implied that the surface density of CTAB bilayers on Au NRs was enhanced in the 1 mM CTAB solution and then reached saturation in the 10 mM CTAB solution. It was also noticeable that CTAB fragment ions corresponding to the loss of methylene groups were observed in the low mass region labeled as F with an increasing concentration of CTAB (Figure 3d). This result further supports our explanation that the surface density of the CTAB bilayers increased with free CTAB because fragmentation mainly took place close to the surface of the Au NRs. All these results suggested that the relative intensity of the mass peaks can reflect the chemical composition on the surface of Au NRs and thus [CTA]^+^ can also be utilized as an internal standard to examine the relative composition of ligand-exchanged Au NRs based on its intensity ratio with the thiol ligands. In addition, a droplet of Au NRs dispersed in water was evaporated, leaving a clear and golden “coffee ring” structure by convection flow toward its edge on a target plate, whereas evaporation of droplets of the Au NRs re-dispersed in 1 mM and 10 mM CTAB solutions led to the thickening and filling of the “coffee ring” with a bluish green color like the color of the suspended Au NRs (Figure 3f). These results indicated that free CTAB improved the colloidal stability of Au NRs even during their evaporation process. After ligand exchange, the LDI-TOF-MS spectra of Au NRs presented mass peaks at *m*/*z* 405, 457, and 693, corresponding to the disulfide cation adducts of AUT [M1 + H]^+^, MUA [M2 + Na]^+^, and TEG [M3 + Na]^+^, respectively (Figure 5a,c,e). Interestingly, the mass peaks of gold cluster ions were not detected from the ligand-exchanged Au NRs (Figure 5). The absence of gold cluster ions can be attributed to the stabilization effect of the thiol ligands through a strong Au-S interaction. This is in agreement with a previous report which showed that the mass signals from gold cluster ions decreased with the density of a thiol ligand [26] and were not detected for Au NPs protected by thiol ligands [19]. We also reported that the mass signals of gold cluster ions were not detected after coating Au NRs with a polydopamine layer [27]. In addition to the disulfide adducts derived from AUT, MUA, and TEG, these compounds have alkyl chains of equal length for the formation of stable self-assembled monolayers (SAMs). Thus, the common fragment ion peaks of their disulfide adducts were strongly detected at *m*/*z* 333 and 361 and were [M1+H-(CH_2_)_3_-(NH_2_)_2_]^+^, [M1+H-CH_2_-(NH_2_)_2_]^+^, [M2+2Na-(CH_2_)_5_-CO_2_H-H-S]^+^, [M2+2Na-(CH_2_)_3_-CO_2_H-H-S]^+^, [M3+H-(CH_2_)_3_-(OCH_2_CH_2_)_6_-2OH]^+^, and [M3+H-CH_2_-(OCH_2_CH_2_)_6_-2OH]^+^, respectively (Figure 5a,c,e). These common fragments were considered together with their corresponding disulfides to obtain the relative intensity ratio of the mass peaks from AUT, MUA, and TEG ligands with respect to CTAB. The relative intensity ratio of the mass peaks from AUT and CTAB (*I_AUT_*/*I_CTAB_*) on ligand-exchanged Au NRs was 1.13 ± 0.26 without additional CTAB (Figure 5a,b). It slightly decreased to 0.97 ± 0.10 in the 1 mM solution of CTAB and increased to 2.14 ± 0.10 in the 10 mM solution of CTAB (Figure 5a,b). This result suggested that an excess of free CTAB facilitated the ligand exchange process of Au NRs with AUT rather than impeded it through competition with AUT. It is important to note that UV–Vis spectroscopy did not present any difference between Au NRs which were modified with AUT in solution with different concentrations of CTAB (Figure 3). The *I_MUA_*/*I_CTAB_* value for the ligand-exchanged Au NRs was 1.21 ± 0.12 without additional CTAB (Figure 5c,d). It substantially decreased to 0.30 ± 0.12 and 0.34 ± 0.09, respectively, after ligand exchange in 1mM and 10 mM CTAB solutions (Figure 5c,d). The decrease of the *I_MUA_*/*I_CTAB_* value with excess free CTAB indicated that ligand exchange of Au NRs with MUA was hindered by free CTAB. This can be ascribed to the opposite charges of CTAB-stabilized Au NRs and MUA ligands, which present permanent positive charges and partial negative charges, respectively. Therefore, the ligand exchange of Au NRs could occur efficiently without additional CTAB owing to the low density of the CTAB bilayer on the surface of the Au NRs, but it resulted in a rapid aggregation of the Au NRs caused by the weakening of the electrostatic repulsion between Au NRs (Figure 3a). Likewise, although excess CTAB lowered the ligand exchange efficiency of Au NRs with MUA because of the increased density of the CTAB bilayer on their surface, CTAB was still required for a moderate ligand exchange with MUA without causing aggregation because free CTAB can compensate for the partial negative charge of MUA ligands and compete with them to suppress to a certain degree the ligand exchange. In the case of TEG, it has neutral and highly hydrophilic properties which cannot provide a high colloidal stability and it thus leads to partial aggregation. Therefore, Au NRs were slightly aggregated (Figure 3), but the mass signal of TEG was well detected without additional CTAB (Figure 5e,f). The *I_TEG_*/*I_CTAB_* value was 0.94 ± 0.15 without additional CTAB, and this value slightly decreased to 0.73 ± 0.05 and 0.78 ± 0.18 in the 1 mM and 10 mM solutions of CTAB (Figure 5e,f). The slight decrease of the *I_TEG_*/*I_CTAB_* value implied that the ligand exchange efficiency of Au NRs with TEG ligands was partially diminished as the concentration of CTAB increased, which is not in agreement with the analytical results of UV–Vis spectroscopy. Considering that the excess CTAB enhanced the surface density of the CTAB bilayers on Au NRs, this slightly decreased *I_TEG_*/*I_CTAB_* was reasonable, and owing to the neutral charge of TEG, the degree of ligand exchange was not significantly affected by the concentration of free CTAB. The error bars in Figure 5b,d,f can be an indicator of the uniformity of the ligand exchange process. As each LDI-TOF-MS spectrum was selectively obtained from the laser-irradiated area (the laser spot size was around 50 μm) of a spot formed by the evaporation of a droplet of the suspended of Au NRs, the error bars in Figure 5b,d, and f might increase when the degree of ligand exchange on Au NRs is not homogeneous. Based on our findings, it is possible to optimize the ligand exchange conditions of Au NRs with thiol derivatives using LDI-TOF-MS analysis. These results clearly show that the shift of the LSPR band provides only limited information about the ligand exchange process, and LDI-TOF-MS can be a powerful and efficient supplement for a successful surface characterization.

## 4. Conclusions

In conclusion, we demonstrated the applicability of LDI-TOF-MS for the surface characterization of Au NRs to explore the degree of ligand exchange with thiol derivatives. Our findings clearly showed that LDI-TOF-MS is a practical and efficient analytical method to investigate the ligand exchange process. Based on the analytical results of LDI-TOF-MS, we revealed that similarly charged ligands are not affected by the concentration of free CTAB, but the ligand exchange process with oppositely charged and neutral ligands is highly dependent on the concentration of free CTAB.

## Data Availability

Data are contained within the article.
